# Combining ability and gene action for flowering, plant height, grain size, and yield in rice (*Oryza sativa L*.) genotypes

**DOI:** 10.3389/fpls.2025.1705322

**Published:** 2026-03-06

**Authors:** Elias Jeke, James Bokosi, Rosemary Murori, Maxwell Darko Asante, Kingsely Masamba

**Affiliations:** 1 Lilongwe University of Agriculture and Natural Resources, Lilongwe, Malawi; 2 Department of Agricultural Research Services, Lifuwu Agricultural Research Station, Salima, Malawi; 3 International Rice Research Institute Kenya, Rice Research Institute (IRRI) – Eastern and Southern Africa (ESA) Africa Regional Office, Nairobi, Kenya; 4 CSIR – Council for Scientific and Industrial Research-Crops Research Institute, Kumasi, Ghana; 5 Department of Plant Resources Development, College of Science and Technology, CSIR – Council for Scientific and Industrial Research, Kumasi, Ghana

**Keywords:** rice breeding, gene action, combining ability, grain yield, correlation

## Abstract

Rice (*Oryza sativa L*.) is one of the most fundamental staple cereal crops feeding more than half of the global population. General Combining Ability (GCA) is the average performance of a genotype across multiple hybrid combinations, mainly due to additive genetic effects while Specific Combining Ability (SCA) is the deviation from expected performance in specific crosses, attributed to non-additive genetic effects such as dominance and epistasis. The aim of the current study was to determine combining ability and gene action of 4 key rice traits such as flowering, plant height, grain size and yield. The study was carried out at Lifuwu Agricultural Research Station – Experimental Fields in Salima District (in Malawi) during the 2024/2025 rainy season in a Randomized Complete Block Design (RCBD) with 3 replications using a total of 15 rice genotypes. Since genetic variance components are not directly observable, crossing methods such as North Carolina Design II (NCD II) was therefore used in the current study to reveal those parameters. Gen stat 19th edition was used for the analysis of majority of the dataset in the current study and Analysis of variance showed significant differences among genotypes, indicating substantial genetic variability across traits. Kudya rice genotype exhibited the highest positive GCA effects (1.015) indicating its strong potential as a grain yield contributor in hybrid combinations. The highest positive SCA effect was exhibited in a cross between Kudya and Kayanjamalo rice germplasm, indicating strong non-additive genetic contribution to yield performance. The highest mean grain yield per plant was recorded in the cross of Kudya × Kayanjamalo (19.0 g), while the lowest was observed in Uwemi × Kilombero (10.3 g). The implication of this study in rice breeding is that superior parents and hybrid combinations for grain yield, earliness to maturity and grain quality were identified for future breeding programmes.

## 1 Introduction

Rice *(Oryza sativa L. 2n = 24)* is the fundamental crop among cereals consumed by two-thirds of the world population (Kahani and Hittalmani, 2015). In Malawi, rice is the second most important cereal crop after maize and is consumed at the rate of 6.53 kg per capita per year. Rice self-sufficiency in Malawi is currently at 98%, despite some rice imports (Ministry of Agriculture (MoA), 2024). The farmers, consumers, and marketers in Malawi generally prefer aromatic, long–slender-grain, and high-yielding cultivars, such as Kilombero and Faya 14 69, among others. However, these cultivars have shortfalls such as late maturation, low yield, lodging, and photosensitivity, leading to a declined productivity. Among several alternatives, the first and high-impact intervention can be accomplished by providing farmers with improved high-yielding, good-quality, and biotitic and abiotic stress-tolerant rice varieties (Mzengeza, 2010; Bagheri and Jelodar, 2010; Bano and Singh, 2019; Kishore et al., 2017).

Therefore, genetic improvement of such cultivars and other existing improved varieties was fundamental in order to increase yield levels whilst maintaining quality traits (Asante, 2017; Pathak Sudhir et al., 2018; Riyanto et al., 2022; Seck et al., 2023; Jeke et al., 2025). The decision was to look for “available traits” and “must have traits” within the product profile in order to come up with unique cultivars that could be readily commercialized and adopted by farmers. The cultivar to be developed must have at least the following traits, so that it could be readily adopted and replace the old varieties: yield ranging 7,000–8,000 kg/ha, more productive tillers, long panicle, early to medium maturity (100–130 days), high milling recovery (>65%), soft when cooked (amylose content of 18%–20%), aromatic (aroma scale of 4-5 on a 5-point scale), tolerant to major biotic and abiotic stresses, and plant height range of 110–125 cm as hypothesized by Li et al. (2014) and Rathod et al. (2016).

Since the parameters are not directly observable, researchers use crossing designs to reveal or dissect such traits (Devi et al., 2018; Nessreen et al., 2021). The agro-morphological traits are applied by breeders in order to estimate genetic parameters such as combining abilities (General Combining Ability and Specific Combining Ability) and gene action (additive variance and dominance variance), heritability, and correlation of characters among parents and their filial generation (Gyawali et al., 2010; Kumar and Gurusubramanian, 2011; Donde et al., 2020). There was lack of information regarding combining abilities, gene action, heritability, and correlation among both improved and local cultivars in order to guide breeders in rice breeding programmes in Malawi. The rationale of the current study was therefore to identify the genetic potential and inheritance pattern of the key agro-morphological traits in the rice genotypes in order to guide the selection of parents and their filial generation for use in further breeding programmes in Malawi. The limitations of the study were the use of a small number of genotypes and that the results were based on advancement trials conducted in one location.

The main objective of the present study was therefore to determine combining abilities and mode of gene action controlling the expression of four key traits, namely, flowering, plant height, grain size, and yield in selected rice (*Oryza sativa L.*) genotypes.

## 2 Materials and methods

### 2.1 Plant materials

The 15 plant materials used in the current study comprised seven parental cultivars and eight F2 progenies. The F2 progenies were advanced from the F1 populations which were developed through hybridization (crossing) of selected parental lines during the 2023/2024 rainy season using the NCD II mating scheme. The parental lines were sourced from Lifuwu Agricultural Research Station as a collection of genotypes from within Malawi’s local landraces, Africa Rice, KAFACI, and the International Rice Research Institute (IRRI) and were all released by the Department of Agricultural Research Services as varieties. The filial generation had generally emanated from cross combinations of the Oryza genus, of the two main species of *O. sativa L.* and *O. glaberrima L.* which geographically comprise landraces, Indica, Japonica, and Tongil type. The basic chromosome number of the two genera (that is, *Oryza sativa L.* and *Oryza glaberrima. L*) is n = 12. The species are either diploid with 2n = 24 chromosomes or tetraploids with 2n = 48 chromosomes. *Oryza sativa L.* is basically an autogamous plant propagating through seeds produced by self-pollination and permit less than a 0.5% natural out crossing. The list of parental and resultant F2 plant materials used in the study are illustrated in **Table 1**.

**Table 1:** List of plant materials used in the study .

No.	Name	Pedigree	Initial source	Type	Special attributes
1	Kayanjamalo	CT 18614-9-3-2-7-2	IRRI	Indica	High yield
2	Kilombero	Landrace	Local collections	Glaberrima	Strong aroma
3	Makafaci	Sahel 328	Africa Rice	Indica	Long grain
4	Mtupatupa	TCG S 10	IRRI	Indica	High tillering
5	NERICA 4	WAB56-104/CG14	WARDA	Indica	Earliness
6	Uwemi (KF18156)	SR32051F1-3-66-1	KAFACI	Tongil	High yield
7	Kudya (KF18055)	HR32080-HB3567-4	KAFACI	Tongil	High yield
8	NERICA 4 × Kilombero	F2	Hybridization	Interspecific recombinant	Earliness + aroma + stress tolerance
9	NERICA 4 × Mtupatupa	F2	Hybridization	Indica hybrid	Earliness, high tillering
10	Uwemi × Mtupatupa	F2	Hybridization	Tongil × Indica hybrid	High yield, high tillering
11	Uwemi × Kilombero	F2	Hybridization	Tongil × Glaberrima hybrid	High yield, aroma, stress tolerance
12	Kudya × Kayanjamalo	F2	Hybridization	Tongil × Indica hybrid	High yield, yield stability
13	Kudya × Mtupatupa	F2	Hybridization	Tongil × Indica hybrid	High yield, high tillering
14	Makafaci × Kilombero	F2	Hybridization	Indica × Glaberrima hybrid	Long grain, aroma, stress tolerance
15	Makafaci × Mtupatupa	F2	Hybridization	Indica hybrid	Long grain, high tillering

IRRI, International Rice Research Institute; KAFACI, Korea – Africa Food for Agricultural Cooperation Initiative; WARDA, West Africa Rice Development Association; NERICA, New Rice for Africa; MAKAFACI, Malawi, Korea -Africa Food and Agriculture Cooperation Initiative.

### 2.2 Experimental site

**Figure 1:**
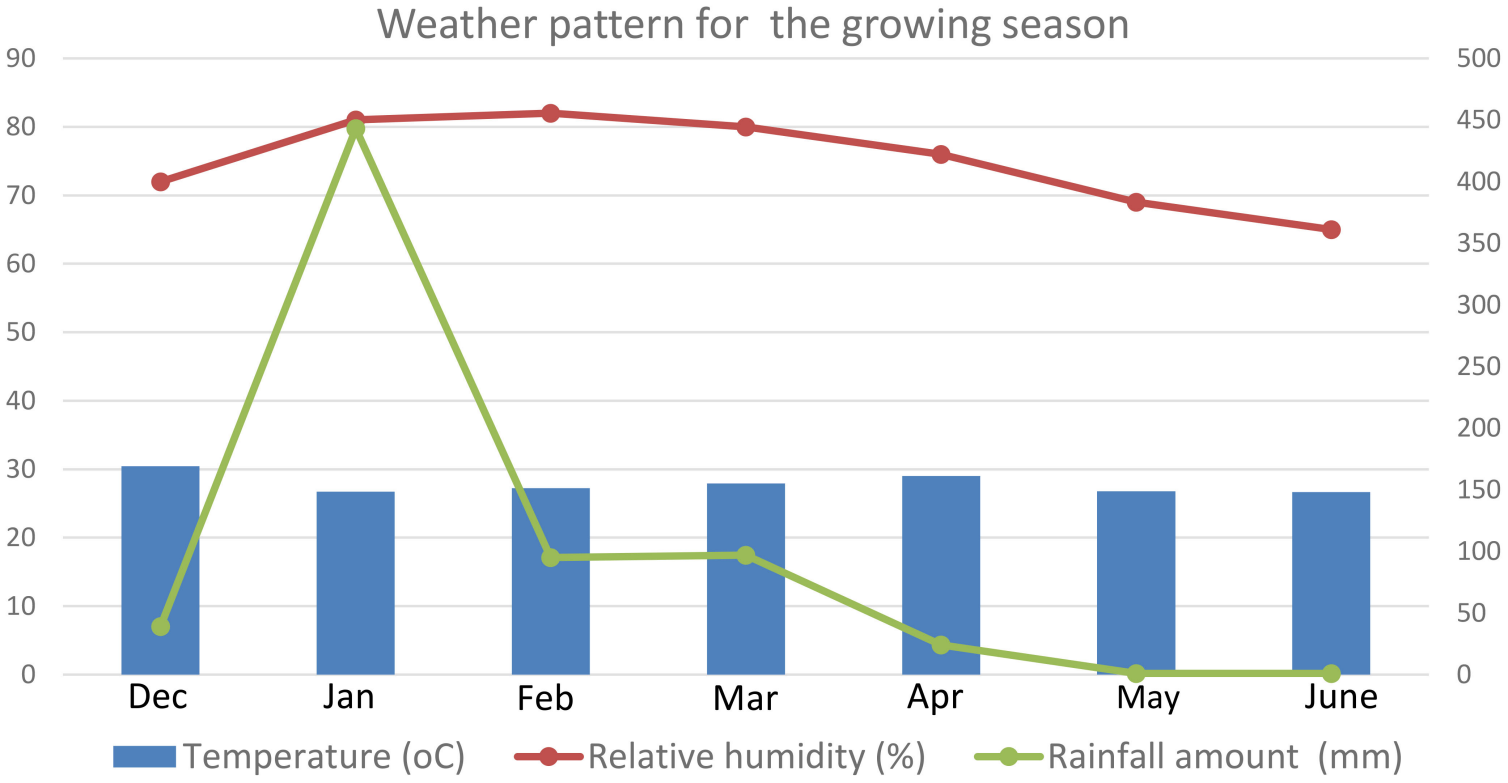
Graph of temperature (°C), relative humidity (%), and rainfall (mm), for Lifuwu site, during 2024/2025.

The crossing activity for development of F1 populations and field experiments of F2 progenies were conducted at Lifuwu Agricultural Research Station in Salima District during the 2023/2024 and 2024/2025 rainy seasons, respectively, under normal growth conditions. The experimental site lies at an altitude of 500 m above sea level (masl), forming part of the great expansive seasonally flooded Katete dambo. It lies at a latitude and longitudinal coordinates of 13.40″ S and 34.35″ E, respectively. The soils for the site could be described as vertisol, 45% clay, comprising low nitrogen and phosphorus content, with a pH range of between 7 and 8. The site receives a unimodal type of rainfall which falls between December and June, and the average amount is 1,230 mm annually. However, during the 2024/2025 season, the site received 698 mm of rainfall with the highest reported in January and the total annual amount was only 56.7% of the sites’ average. Supplementary water for irrigation in order to meet the crop water requirement was pumped from Lake Malawi using electric energy through pipes which were directly connected onto the experimental fields. The mean annual temperatures ranged between a minimum of 16°C to a maximum of 28°C, and relative humidity of between 65% and 82% was experienced during the same season at the Lifuwu site (**Figure 1**).

### 2.3 Experimental design

There are many mating designs used in rice breeding to aid in enhancing various traits such as grain yield and quality, among other attributes (Abd-El-Aty et al., 2023; Kumari et al., 2022; Dianga et al., 2020; Singh et al., 2024; Ochigbo, 2023. However, the North Carolina Design II mating scheme (NCD II) has greater precision and is more applicable to self-pollinated crops such as rice for obtaining information about combining ability, gene action, and heritability (Abdel-Moneam et al., 2015; Devi, 2017; Soni et al., 2021). In North Carolina Design II (NCD II), each germplasm of a group of males is mated to each germplasm of a group of females (Comstock and Robinson, 1952; Muthoni and Shimelis, 2020).

The crossing programme in the current study was therefore constituted using the North Carolina Mating Design II (NCD II) during the 2023/2024 rainy season and involved different sets of four female and three male parents of Indica, Japonica, Glaberrima, and Tongil-types (**Table 1**). The choice of parents for crossing was therefore based on both yield and quality traits such as grain size, earliness to maturity, tillering ability, and aroma. During cross breeding, two distinct sets of parental lines were interchangeably used whereby each male parent was crossed with every female parent from a separate group. Since it was not easy to obtain adequate F1 seeds through hand emasculation, the F1 seeds were therefore multiplied (selfed) to produce F2 seeds in the 2024 irrigated conditions. It was easy to produce a large quantity of F2 seeds from the multiplication and advancement of the F1 generation.

The parental lines along with their F2 progenies were therefore grown in the field conditions during the 2024/2025 rainy season in order to estimate combining abilities and other genetic parameters. The rice genotypes were planted in a randomized complete block design (RCBD) with three replications. The nursery was prepared in December 2024, and transplanting into the field plots was done in January 2025 before the seedlings reached an age of 21 days after emergence. Each rice genotype was transplanted in a plot consisting of 5 m × 0.4 m, thus a space of 20 cm between rows and 20 cm between plant stations as well. In this regard, each plot comprised 3 rows and 26 hills thus giving a total of 78 hills. A space of 1 m and 0.4 m was maintained to separate one replicate from another and between plots, respectively. Blanket application of fertilizer was maintained at the total rate of 180 kg/ha, split into 120 kg/ha for NPK1S6Zn and 60 kg/ha urea using the broadcasting application method. The basal fertilizer was applied immediately after transplanting (same day), and the top-dressing fertilizers application was done 45 days later. No other chemicals apart from fertilizers were used in the trial. Weeding was done two times using hands, and supplementary water was applied as soon as cracks started to form in the experimental field.

### 2.4 Data collection

Data were collected from the composite sample of five plants from the middle row, and the targeted four key traits were grain yield/plant, days to 50% flowering, plant height, and grain size (thus, grain length, grain width, grain length/width ratio). The additional trait such as 100 grain weight was also measured in the course of handling the samples in the laboratory and had been reported accordingly. The number of days to heading was counted from the date of seedling emergency to the day when 50% tillers of each sampled plant had exposed panicles. Plant height was recorded in centimetres from the surface of the soil to the tip of the panicle. Grain yield per plot was generally determined by weighing a threshed sample of five plants (hills) and converting it per plot on rough (paddy) rice at 14% moisture content; however, what had been reported in this paper is grain yield per plant in grams. Grain size (grain length, grain width, and grain length/width ratio or shape) was estimated in millimetres (except shape) using a Vernier calliper. Grain length was measured using a Vernier calliper in millimetres as the distance from the base of the lowermost sterile lemma to the tip (apiculus) of the fertile lemma or palea as stipulated in the standard evaluation system for rice, International Rice Research Institute (IRRI) (2013). Grain width (mm) was measured using a Vernier calliper as the distance across the fertile lemma and palea at the widest point. In the case of awned varieties/lines, the grain was measured to a point comparable with the tip of the apiculus.

### 2.5 Data analysis

The F2 population data were subjected to the general analysis of variance (ANOVA) technique appropriate for randomized complete block design and REML for the analysis of combining abilities and other genetic parameters by GenStat,19^th^ Edition. The recommended ANOVA model for the randomized complete block design at one location and in the 1 year of testing was as described in Equation 1:

(1) Yij=μ+ai+βj+Єij,

where: Y_ij_ is the observed value of the i^th^ genotype for the j^th^ replicate (where i =1 to 7) and (j = 1 to 3); µ is the grand mean; α_i_ is the treatment effect for the i^th^ genotype, with the genotypes assumed to be random; β_j_ is the block effect for the j^th^ block; and Єij is the random error associated with the Y_ij_ experimental unit representing the unexplained variation in the data. Since the North Carolina Mating Design II (NCD II) mating scheme was used, the following ANOVA was therefore appropriate and adopted accordingly (**Table 2**).

**Table 2:** ANOVA table for North Carolina Design II.

Source of variation	Df	Expected mean square	Variance components
Female parents	(p -1)	MS p	σ^2^_SE_ + rσ ^2^ pq + rq σ^2^ p
Male parents	(q-1)	MS q	σ^2^_SE_ + rσ ^2^ pq + rp σ^2^ q
Female × male	(p -1) (q-1)	MS pq	σ^2^_SE_ + rσ ^2^ pq
Standard error	pq (r-1)	MS_SE_	σ^2^_SE_

p, female parents; q, male parents; MS p, mean square for female parents; MS q, mean square for male parents; Df, degrees of freedom; MS_SE_, standard error of the mean square; and σ^2^ SE, standard error of the variance.

The analysis of general combining ability (GCA) and specific combining ability (SCA) are vital for determination of additive and non-additive genetic effects on target characters. In the current study, the variation in the crosses for the analysis of combing ability was partitioned into general combining ability (GCA) and specific combining ability (SCA). The adopted genetic model was therefore presented in Equation 2:

(2) $$Y_{pqk}=\mu +GCA_{p}+GCA_{q}+SCA_{pq}+rk+\in_{pqk}$$

Where Y is the hybridization mean between *p* and q parents (such that *p* is the female parent and q is the male parent); GCAp is the general combining ability of the female parent (p); GCAq is the general combining ability of male parent (*q*); SCApq is the specific combining ability between female parent (*p*) and male parent (q); rk is the replication effect; and ∈_pq_ is the error term or residual associated with the Ypq experimental units.

The specific combining ability (SCA) and general combining ability (GCA) effects were calculated using the formula reported by Yu et al. (2020) as follows:

Equation 3:

(3) $$\begin{array}{l} g_{i}={\bar{y}}_{i}-\bar{y}\\ s_{ij}=y_{ij}-\bar{y}-g_{i}-g_{j} \end{array}$$

where gi and gj are the GCA effects for the ith and jth germplasms, respectively; sij is the SCA effect for the ijth F2 progeny; yij is the trait value for the ijth F2 progeny; $\bar{y}$ is the overall mean**;** and ${\bar{y}}_{i}$ is the average of the F2 progenies among the ith germplasm hybridized with a series of parents.

In order to determine whether the effects were significant or not, a comparison between each effect to the standard error (SE) was undertaken and the t-test was subsequently applied using the following formula, for Equation 4:

(4) $$t=\frac{Effect}{Standard\;Error}$$

If the t-value was greater than (>) the critical (p-value), and then the GCA was regarded as significant and vice versa for smaller t-values. Related variance components were calculated using the formulae highlighted in **Table 3**.

**Table 3:** Variance components usage.

Variance component	Symbol	Common formula	Measured value	Importance in rice breeding
Additive	σ^2^A	σ^2A^ = 2 x (σ^2^M + σ^2^F)	Breeding value	Parental allele effects
Dominance	σ^2D^	σ^2D = 4 x^ (σ^2^MF)	Dominance deviation	Allele interaction
Degree of dominance	d^2^	D = √σ^2D/^σ^2^A	Ration of dominance and additive	Trait expression
Interaction	σ^2^I	σ^2^I = σ^2^P/σ^2^G + σ^2^E	Interaction deviation	Aid selection
Environmental	σ^2^E	σ^2^E = σ^2^P– (σ^2^A+σ^2D^)	Environmental deviation	Stability assessment
Phenotypic	σ^2^P	σ^2^P = σ^2^G+σ^2^E +	Phenotypic value	Effective selection
Genotypic	σ^2^G	σ^2^G = (σ^2^A + σ^2^D)	Genotypic value	Effective selection
Heritability (narrow)	h^2^	h^2^_=_ σ^2^G/σ^2^P	Phenotypic variance	Prediction

## 3 Results

### 3.1 Trait variation

There was no significant general combining ability variance in both the male and female parents mainly for the number of days to 50% flowering, plant height, and number of panicles per plant at 5% probability level. Analysis of variance (ANOVA) for plant height (PH) and other traits among F_2_ progenies and their parental germplasm is summarized in **Table 4**. The interaction between female and male parents was statistically significant (*p* < 0.05), indicating the presence of genetic variability due to specific parental combinations. However, the main effects of female and male parents were not significant, for the plant height trait. The general and specific combining ability variances for panicle length in the female parents and their F2 progenies revealed that they were significant among them (mean square = 14.729, p < 0.05) and the interaction effect (mean square =10.156, p < 0.05), respectively. The analysis of variance for number of panicles per plant (NPP) among F_2_ progenies and their parents showed statistically no significant variations (*p* > 0.05). This implies that the observed variation in this trait among the F_2_ progenies was not significantly influenced by the parental genotypes or their combinations. The analysis of variance for grain length (GL) among F_2_ progenies and their parents revealed no statistically significant differences (*p* > 0.05). This means that the differences in grain length observed among the F_2_ progenies were not significantly influenced by the parental genotypes or their combinations.

**Table 4:** Mean square from analysis of variance for four key traits of F2 progenies and their parents .

Source of variation	Df^▪^	Trait and corresponding mean square (MS)							
		DTF 50%	PH (cm)	PL	NPP	GL (cm)	GW (cm)	GS	GYPP (g)
Female parents	3	21.44^ns^	192.38 ^ns^	14.729*	11.704 ^ns^	0.215 ^ns^	0.148*	0.553 ^ns^	9.94^ns^
Male parents	2	32.06 ^ns^	240.09 ^ns^	1.938^ns^	6.838 ^ns^	0.341^ns^	0.005 ^ns^	0.063 ^ns^	44.49*
Female × male	7	23.33 ^ns^	214.34*	10.156*	8.042 ^ns^	0.210 ^ns^	0.089*	0.355 ^ns^	21.54^ns^
Error	14	12.14	42.45	1.814	6.485	0.375	0.027	0.167	12.84

*Significant at the 5% level, **significant at 1% level, ^ns^, non-significant; Df, degree of freedom; DTF, days to 50% flowering; PH, plant height; PL, panicle length; NPP, number of panicles per plant; GL, grain length; GW, grain width; GS, grain shape; GYPH, grain yield per plant.

Significant differences were exhibited among female parents (*p* < 0.05) for grain width, suggesting that maternal lines contributed notably to differences for this trait. In addition, the interaction between female and male parents was also significant (*p* < 0.05), suggesting that specific parental combinations influenced the expression of this trait. However, the effect of male parents alone was not statistically significant as evidenced by the critical value (*p* > 0.05). The analysis of variance for grain shape among F_2_ progenies and their parents is illustrated in **Table 4**. The sources of variation including female and male parents, or their interaction exhibited that they were not statistically significant (*p* > 0.05).

The analysis of variance (ANOVA) for grain yield per plant (GYPP) revealed significant differences among male parents (p < 0.05), confirming the presence of substantial genetic variation within this category (**Table 4**). However, no significant difference was exhibited from the interaction between female and male parents (p > 0.05), suggesting limited variation or absence of differential combining ability among the female lines for this trait.

#### 3.1.1 ANOVA for the (F2 s)

**Table 5** provides a summary of the mean trait values for the interaction of female and male parents in F2 progenies for traits such as days to 50% flowering, plant height, panicle length, number of panicles per plant, grain size (grain length, grain width, grain shape), and grain yield per plant. The number of days to 50% flowering (DTF) ranged from 111 (Kudya × Mtupatupa) to 118 (Uwemi × Kilombero), and a mean of 115 days among the various cross combination. Furthermore, Makafaci × Mtupatupa and NERICA 4 × Kilombero exhibited higher-than-average in terms of number of days to reach 50% flowering.

**Table 5:** Analysis of variance (ANOVA) for the F2 progenies .

Cross combination	DTF 50%	PH (cm)	PL (cm)	GL (mm)	GW (mm)	GS	NPP	GYPP (g)
NERICA 4 × Kilombero	117	80	26.3	9.3	2.4	3.9	10	14.5
NERICA 4 × Mtupatupa	112	77	23.8	9.1	2.6	3.5	12	13.9
Uwemi × Mtupatupa	114	89	22.8	9.2	2.6	3.6	9	14.9
Uwemi × Kilombero	118	73	22.9	8.8	2.8	3.2	8	10.3
Kudya × Kayanjamalo	112	96	23.4	9.7	2.5	3.9	9	19.0
Kudya × Mtupatupa	111	78	23.7	9.2	2.5	3.6	10	12.1
Makafaci × Kilombero	114	93	24.6	9.4	2.4	3.9	10	14.8
Makafaci × Mtupatupa	117	90	28.1	9.3	2.2	4.3	13	11.4
Mean	115	85	24.4	9.2	2.5	3.7	10	13.9
SE	2.011	6.5	1.35	0.35	0.10	0.24	2.5	2.07
LSD	6.101	11.4	2.36	1.07	0.29	0.72	4.5	6.27
F-prob	0.141	0.005	0.003	0.700	0.028	0.109	0.345	0.19
CV (%)	3.0	7.7	5.5	6.7	6.6	10.9	25.1	25.8

SE, standard error; LSD, least significant difference; CV, coefficient of variation; DTF, days to flowering at 50%; PH, plant height; PL, panicle length; NPP, number of panicles per plant; GL, grain length; GW, grain width; GS, grain shape; GYPP, grain yield per plant.

There were significant differences exhibited for plant height among the studied rice genotypes across combinations (F-prob = 0.005) at 0.05% critical value. The highest plant height was attained in the cross combination of Kudya × Kayanjamalo (96 cm), whereas the lowest was in Uwemi × Kilombero (73 cm). The overall mean plant height across all crosses was 85 cm, with a coefficient of variation (CV) of 7.7%, suggesting moderate variability. The least significant differences (LSD) magnitude of 11.4 cm provides a threshold for determining significant variations among the cross combinations.

Significant differences were attained with an F-probability value (0.003) for panicle length across combinations. The results indicate that Makafaci × Mtupatupa cross exhibited the highest panicle length (28.1 cm), whereas Uwemi × Mtupatupa combination had the lowest panicle length (22.8 cm). The overall mean panicle length across all combinations was 24.4 cm, with a standard error (SE) of 1.35 cm. The least significant difference (LSD) was calculated at 2.36 cm, indicating the minimum difference required for significance among cross-combinations. In addition, the coefficient of variation (CV) was 5.5%, indicating relatively low variability among the tested combinations.

There were no significant differences in terms of number of panicles per plant as evidenced by an F-probability of 0.345 at 0.05% critical value. The mean number of panicles per plant was 10, and the coefficient of variation (CV) of 25.1% was attained among the studied rice genotypes. The grain length of F2 progenies ranged from 8.8 mm (Uwemi × Kilombero) to 9.7 mm (Kudya × Kayanjamalo), with an overall mean of 9.2 mm. However, the variations among the cross combinations were not statistically significant (*p* = 0.700), as illustrated by the ANOVA. The relatively low coefficient of variation (CV = 6.7%) suggests good experimental precision.

Grain width (GW) among the F2 progenies exhibited significant variation (F-prob = 0.028) across the various cross combinations. The mean grain width was 2.5 mm, and the range was from 2.2 mm (Makafaci × Mtupatupa) to 2.8 mm (Uwemi × Kilombero). The analysis of variance revealed a statistically significant difference among the crosses (F-prob = 0.028), indicating that genetic background had a notable effect on grain width. The least significant difference (LSD) at 5% was 0.29 mm, and the coefficient of variation (CV) was relatively low at 6.6%, suggesting good experimental precision. Grain shape (GS) among the F2 progenies varied across the different cross combinations, with values ranging from 3.2 (Uwemi × Kilombero) to 4.3 (Makafaci × Mtupatupa), and a mean of 3.7. Although numerical differences were observed, the ANOVA results revealed that these differences were not statistically significant at the 5% level (F-prob = 0.109). The least significant difference (LSD) was 0.72, and the coefficient of variation (CV) was 10.9%, suggesting moderate variability.

Grain yield per plant (GYPP) differed across the studied rice genotypes including the F2 populations. The highest mean yield was recorded in the cross of Kudya × Kayanjamalo (19.0 g), whereas the lowest was observed in Uwemi × Kilombero (10.3 g). The overall mean yield of the studied F2 progenies was 13.9 g. The analysis of variance exhibited no statistically significant differences among the progenies (F-prob = 0.19); however, biological variations were evident, with a coefficient of variation (CV) of 25.8%. The standard error (SE) was 2.07 g, and the least significant difference (LSD) at the 5% level was 6.27 g.

**Figure 2:**
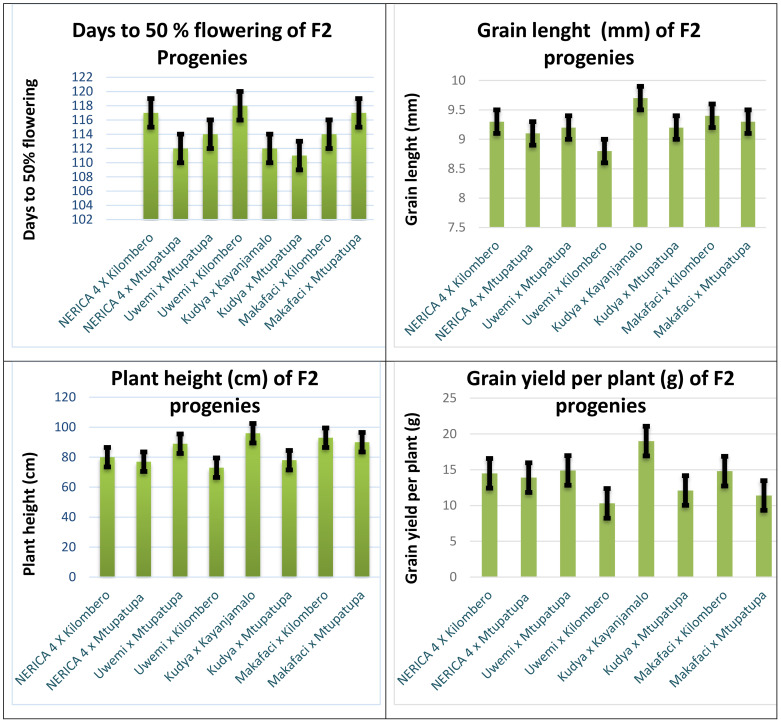
Graphical presentation of four key traits for the eight F2 progenies.

**Figure 2** shows a graphical presentation of the four key targeted traits for this study, namely, days to 50% flowering, plant height (cm), grain length (cm), and grain yield per plant (g). The earliest number of days to reach 50% flowering was revealed in a cross involving Kudya × Mtupatupa, and the latest was obtained in the Uwemi × Kilombero cross, respectively, as evidenced by the bar-magnitude. Plant height was the shortest in the cross involving Uwemi × Kilombero, and the tallest was revealed in Kudya × Kayanjamalo. The shortest grain length was recorded in a cross combination of Uwemi × Kilombero, and the longest was in Kudya × Kayanjamalo. The lowest-yielding F2 progeny was Uwemi × Kilombero, and the highest yielder was depicted in a cross combination of Kudya × Kayanjamalo.

### 3.2 Combining ability

#### 3.2.1 General combining ability effects

The general combining ability (GCA) effects for days to 50% flowering among the four female parents illustrated variation, ranging from −6.000 (Kudya) to 3.333 (NERICA 4). The cultivars such as Uwemi and Kudya showed negative GCA effects, whereas MAKAFACI and NERICA 4 showed positive values, suggesting variations in their influence on this trait. Kudya rice cultivar had the most pronounced negative effect (−6.000), suggesting potential early flowering behaviour, whereas NERICA 4 had the highest positive effect (3.333), indicating a stay-green trait for delayed flowering (**Table 6**).

**Table 6:** General combining ability effects for the key traits of four female parents.

Parent name	DTF 50%	PH	PL	GL	GW	GS	NPP	GYPP
MAKAFACI	0.001	0.001	0.001	0.001	0.001	0.001	0.001	0.001
NERICA 4	3.333	−12.472	1.656	−0.141	0.218	−0.426	0.109	1.064
Uwemi	−3.667	−0.254	−5.264	−0.345	0.382	−0.741	−4.556	−0.499
Kudya	−6.000	−11.584	−4.323	−0.193	0.298	−0.568	−3.628	1.105

DTF, days to flowering at 50%; PH, plant height; PL, panicle length; NPP, number of panicles per plant; GL, grain length; GW, grain width; GS, grain shape; GYPP, grain yield per plant.

MAKAFACI variety exhibited a near-neutral effect (0.001) magnitude for plant height, indicating a minimal influence on the inheritance of this trait. NERICA 4 and Kudya showed a strong negative GCA effect of (−12.472) and (−11.584), respectively, suggesting that these parents significantly reduce plant height in crosses whereas the Uwemi variety exhibited a slight negative effect (−0.254), as displayed in **Table 6**.

Among the four female parents used in the hybridization, NERICA 4 indicated the highest positive GCA effect (+1.656), suggesting a strong genetic potential for enhancing panicle length. MAKAFACI showed a nearly neutral effect (+0.001), suggesting minimal influence on panicle length in hybrid combinations. On the other hand, Uwemi and Kudya displayed negative GCA effects (−5.264 and −4.323, respectively), suggesting a decline in panicle length when these parents are used in breeding programmes.

MAKAFACI exhibited a near-neutral GCA effect (0.001) in terms of number of panicles per plant, implying its minimal genetic influence on this trait. NERICA 4 shows a slightly positive GCA effect (0.109), indicating a modest enhancement in panicle production. Uwemi and Kudya demonstrate substantial negative GCA effects of −4.556 and −3.628, respectively), suggesting that progeny derived from these parents may exhibit significantly reduced panicle contribution. Makafaci exhibited a near-zero GCA effect (0.001), indicating a neutral contribution to grain length in its progenies. In contrast, NERICA 4, Uwemi, and Kudya showed negative GCA effects of −0.141, −0.345, and −0.193 mm, respectively, implying that these parents may lead into a declined grain length when used in hybrid combinations. These results imply that Makafaci may be a suitable parent for improving grain length in breeding programmes, whereas the other three may contribute to shorter grains in their progenies (**Table 6**). All parents showed positive GCA effects, indicating a general tendency to increase grain width in their progenies. Uwemi recorded the highest GCA effect (0.382), followed by Kudya (0.298) and NERICA 4 (0.218), suggesting that these parents are favourable contributors to wider grains. MAKAFACI showed a near-zero GCA effect (0.001), suggesting a negligible influence on this trait. These results indicate that Uwemi, Kudya, and NERICA 4 are promising female parents for improving grain width in breeding programmes, with Uwemi being the most effective general combiner for this trait.

Makafaci exhibited a near-zero GCA effect (0.001) for grain shape, indicating a neutral contribution in its progenies for this trait. In contrast, NERICA 4 (−0.426), Kudya (−0.568), and Uwemi (−0.741) showed negative GCA effects, suggesting a tendency to contribute in declined grain shape values when used as female parents. The analysis of general combining ability (GCA) for grain yield per plant (GYPP) revealed variation among the four female parents (**Table 6**). Kudya exhibited the highest positive GCA effect (1.105), indicating its strong potential as a contributor to grain yield in hybrid combinations. NERICA 4 also showed a favourable GCA effect (1.064), whereas MAKAFACI had a near-zero effect (0.001). In contrast, Uwemi displayed a negative GCA effect (−0.499), suggesting a comparatively weaker contribution to yield performance.

The GCA effects for the male parents in the present study for the target traits such as days to flowering, plant height, panicle length, number of panicles per plant, grain size (grain length, grain width, and grain shape), and grain yield per plant are shown in **Table 7**. The GCA effect for number of days to 50% flowering for Kayanjamalo (0.001) value is near-zero, and this implies that this parent has a very minimal effect on its progenies. Kilombero (−4.333) had a negative GCA value, indicating that this parent contributes to earlier flowering in offspring, despite it being a known late flowering cultivar. Furthermore, Mtupatupa (−1.000) had also a negative GCA, although less strongly than Kilombero, and this means that this parent contributes to earlier flowering but at a moderate level compared with Kilombero.

**Table 7:** General combining ability effects for the key traits of three male parents.

Parent name	DTF 50%	PH	PL	GL	GW	GS	NPP	GYPP
Kayanjamalo	0.001	0.01	0.001	0.001	0.001	0.001	0.001	0.001
Kilombero	−4.333	−15.09	−3.068	−0.565	0.134	−0.411	−2.722	−7.025
Mtupatupa	−1.000	−18.23	0.345	−0.504	0.067	−0.277	0.656	−6.853

DTF, days to flowering at 50%; PH, plant height; PL, panicle length; NPP, number of panicles per plant; GL, grain length; GW, grain width; GS, grain shape; GYPP, grain yield per plant.

There was a negligible effect on plant height for Kayanjamalo (0.01), suggesting that it neither significantly increases nor reduces height in its offsprings. Kilombero (−15.09) exhibited a negative GCA effect, implying that it contributes to shorter plants in hybrid combinations despite it being phenotypically tall. A stronger negative GCA effect was attained in Mtupatupa (−18.23), indicating that it has a more pronounced height-reducing influence in its progeny.

The small but positive effect for Kayanjamalo (0.001) suggests a negligible additive genetic influence on panicle length in hybrid progenies. Mtupatupa attained a GCA effect of 0.345, suggesting a relatively stronger additive genetic contribution to panicle length. This implies that Mtupatupa could be a favourable male parent in breeding programmes aimed at increasing panicle size, which is often linked to yield potential. On the other hand, Kilombero exhibited a markedly negative GCA effect (−3.068) for this trait, implying that its genetic contribution leads to a declined panicle length.

Kayanjamalo exhibited a negligible GCA effect of 0.001, suggesting that this parent has a small influence both negatively and positively for the number of panicles per plant in its hybrid progenies. Kilombero had a significant negative GCA effect of −2.722, indicating that crosses comprising this parent lead to a declined number of panicles per plant in the progeny. This suggests that Kilombero may not be ideal for improving panicle number. Mtupatupa revealed a positive GCA effect of 0.656, suggesting that it contributes favourably to increasing the number of panicles per plant in its progeny.

Kayanjamalo exhibited a near-zero GCA effect (0.001 mm), indicating a neutral influence on grain length in its progenies. On the other hand, Kilombero (−0.565) and Mtupatupa (−0.504) showed negative GCA effects, indicating that these parents tend to reduce grain length when used in hybridization. Among the male parents, Kilombero had the most negative GCA effect, indicating the least favourable contribution to this trait. These findings suggest that Kayanjamalo may be a more suitable male parent for maintaining or improving grain length in breeding programmes. The general combining ability (GCA) effects for grain width was positive for all the three parents. Kilombero recorded the highest GCA effect (0.134), followed by Mtupatupa (0.067), whereas Kayanjamalo showed a near-zero effect (0.001), suggesting a minimal influence. These results suggest that Kilombero is the most promising male parent for improving grain width in breeding programmes, whereas Kayanjamalo may have a neutral effect on this trait. Kayanjamalo exhibited a near-zero GCA effect (0.001), indicating a neutral contribution to grain shape in its progenies. On the other hand, Kilombero (−0.411) and Mtupatupa (−0.277) revealed negative GCA effects, suggesting a tendency to reduce grain shape when used as male parents. These results suggest that Kayanjamalo may be a more suitable male parent for enhancing or maintaining grain shape in breeding programmes.

The GCA effect for grain yield per plant for Kayanjamalo was near zero (0.001), indicating a negligible but positive contribution to grain yield. In contrast, both Kilombero and Mtupatupa showed negative GCA effects of −7.025 and −6.853, respectively, suggesting relatively poorer combining ability for this trait compared with Kayanjamalo (**Table 7**). These results highlight Kayanjamalo as the most promising male parent for improving grain yield per plant in subsequent breeding efforts.

#### 3.2.2 Specific combining ability for the F2 progenies

The specific combining ability of the F2 progenies for number of days to 50% flowering, plant height, panicle length, number of panicles per plant, grain length, grain width, grain shape, and grain yield pe plant is shown in **Table 8**. Generally, the number of Days to 50% Flowering (DTF 50%) is vital in rice breeding as it helps in making inferences for early or late-flowering varieties. The positive SCA of 8.00 obtained in a cross involving Uwemi × Kilombero implies delayed flowering, which may be a vital trait for certain climate conditions where later flowering of the genotypes contributes towards improved yield. The SCA value of −9.00 attained in a cross involving NERICA 4 × Mtupatupa indicates an early flowering attribute, which can be useful for short-season adaptation cultivars. Furthermore, a close-to-zero value (0.001) exhibited in a majority of crosses suggest minimal influence of specific combining ability on flowering time, implying that these crosses have stable flowering behaviour across different parental combinations.

**Table 8:** Specific combining ability effects for the key traits of F2 progenies.

Cross combination	DTF 50%	PH	PL	NPP	GL	GW	GS	GYPP
NERICA 4 × Kilombero	0.001	0.001	0.001	0.001	0.087	−0.168	0.301	−0.453
NERICA 4 × Mtupatupa	−9.00	−0.086	−5.896	−1.961	−0.163	−0.024	−0.093	−1.005
Uwemi × Mtupatupa	0.001	0.001	0.001	0.001	0.001	0.001	0.001	0.001
Uwemi × Kilombero	8.00	−19.350	3.506	2.864	−0.483	0.184	−0.419	−4.585
Kudya × Kayanjamalo	0.001	0.001	0.001	0.001	0.445	−0.092	0.307	4.041
Kudya × Mtupatupa	0.001	0.001	0.001	0.001	−0.058	−0.026	0.030	−2.812
MAKAFACI × Kilombero	0.001	0.001	0.001	0.001	0.128	−0.186	0.342	−0.035
MAKAFACI × Mtupatupa	0.001	0.001	0.001	0.001	0.078	−0.394	0.719	−3.551

DTF, days to flowering at 59%; PH, plant height; PL, panicle length; NPP, number of panicles per plant; GL, grain length; GW, grain width; GS, grain shape; GYPP, grain yield per plant.

Several crosses exhibited negligible positive SCA values (0.001 cm), such as NERICA 4 × Kilombero, Uwemi × Mtupatupa, Kudya × Kayanjamalo, Kudya × Mtupatupa, MAKAFACI × Kilombero, and MAKAFACI × Mtupatupa. These marginal values suggest that these specific combinations do not significantly enhance plant height beyond general genetic expectations.

The negative SCA values were attained in NERICA 4 × Mtupatupa (−0.086) and Uwemi × Kilombero (−19.35) with the latter showing a considerably strong decline in plant height. This suggests strong non-additive genetic effects that suppress height expression, possibly due to incompatibility or the influence of recessive alleles.

NERICA 4 × Mtupatupa exhibited the strongest negative SCA effect (−5.896), suggesting poor genetic complementarity for panicle length.

Uwemi × Kilombero exhibited the highest positive SCA effect (3.506), implying a strong, favourable interaction between the parental genotypes. Other combinations such as NERICA 4 × Kilombero, Uwemi × Mtupatupa, Kudya × Kayanjamalo, Kudya × Mtupatupa, MAKAFACI × Kilombero, and MAKAFACI × Mtupatupa) exhibited near-zero effects (0.001), indicating negligible impact on panicle length beyond parental expectations.

The positive SCA values suggest a favourable interaction between parental lines, leading to an increase in the number of panicles per plant. The highest positive SCA effect is Uwemi × Kilombero (2.864), suggesting a strong hybrid vigour that enhances panicle production. The negative SCA value effect suggests that the specific parental combination is less effective in strengthening the number of panicles. NERICA 4 × Mtupatupa (−1.961) indicates the strongest negative impact, potentially due to unfavourable interactions or genetic incompatibilities. Several cross combinations such as NERICA 4 × Kilombero, Kudya × Kayanjamalo, and MAKAFACI × Kilombero, among others, indicate minimal SCA effects (~0.001) in terms of number of panicles per plant (**Table 8**). These combinations may contribute little variation in panicle number and could suggest stable but unremarkable genetic interactions. The cross Kudya × Kayanjamalo exhibited the highest positive SCA effect (0.445), suggesting potential for grain length improvement. However, the Uwemi × Kilombero combination recorded the most negative SCA effect (−0.483), implying less favourable genetic interaction for this trait. The Uwemi × Kilombero cross produced the highest positive SCA effect (0.184), suggesting a promising combination for wider grain development. In contrast, MAKAFACI × Mtupatupa exhibited the most negative SCA effect (−0.394), suggesting limited potential for grain width improvement. The MAKAFACI × Mtupatupa cross exhibited the highest positive effect (0.719), suggesting strong potential for grain shape improvement. Negative SCA effects were observed in NERICA 4 × Mtupatupa (−0.093) and Uwemi × Kilombero (−0.419), indicating weaker combining ability for this trait in those hybrids (**Table 8**).

The Specific Combining Ability analysis for Grain Yield Per Plant demonstrated considerable variability among the F2 progenies. The cross between Kudya and Kayanjamalo exhibited the highest positive SCA effect (4.041), indicating strong non-additive genetic contributions to yield performance. Conversely, Uwemi × Kilombero (−4.585) and MAKAFACI × Mtupatupa (−3.551) recorded the most negative SCA effects, suggesting limited hybrid vigour from those combinations. Notably, Uwemi × Mtupatupa (0.001) exhibited near-zero SCA, implying an additive or neutral combining effect (**Table 8**).

### 3.3 Genetic analysis

The mode of gene action in this study comprised variance components, such as phenotypic variance, additive variance, dominance variance, environmental variance, and genetic variance as well as calculation of heritability and degree of dominance (**Table 9**).

**Table 9:** Estimation of mode of gene action and trait heritability in 8 F2 progenies.

Trait	Magnitude of mode of gene action						
	Ns (h^2)^ ± SE (%))	σ^2^P	σ^2^A	σ^2^D	σ^2^E	σ^2^G	(d^2^)
DTF 50%	0.24 **±** 0.16	23.26	5.58	5.58	12.1	11.2	1.00
PH	0.03 **±** 0.12	280.20	8.57	229.18	42.45	237.76	5.15
PL	0.14 ± 0.10	14.97	2.02	11.11	1.84	13.15	2.33
NPP	0.07 ± 0.11	9.175	0.614	2.075	6.48	2.687	1.82
GL	0.40 ± 0.51	0.82	0.33	0.28	0.21	0.61	0.94
GW	0.00	0.12	0.00	0.08	0.03	0.08	0.00
GYPP	0.39 ± 0.23	78.25	30.61	34.80	12.84	65.41	1.05

Ns (h^2^), narrow sense heritability; σ^2^P, phenotypic variance; σ^2^A, additive variance; σ^2^D, dominance variance; σ^2^E, environmental variance; σ^2^G, genotypic variance; d^2^, degree of dominance;DTF, days to flowering at 50%; PH, plant height; PL, panicle length; NPP, number of panicles per plant; GL, grain length; GW, grain width; GYPP, grain yield per plant.

The number of days to 50% flowering (DTF) in the F2 rice progenies exhibited a low to moderate narrow-sense heritability of 0.24 ± 0.16, suggesting modest contribution of the additive genetic effects to the phenotypic variation. The additive variance (σ²A) and dominance variance (σ²D) were of same magnitude of 5.58 each, implying that both additive and dominance gene actions are equally important in the inheritance of this trait. The degree of dominance (d²) was 1.00, indicating complete dominance. The genetic variance (σ²G) of 11.20 accounted for nearly half of the phenotypic variance (σ²P), 23.26, whereas the environmental variance (σ²E) of 12.10 was also substantial, reinforcing the influence of environmental factors on heading time (**Table 9**).

Plant height (PH) in the F2 rice progenies showed very low narrow-sense heritability of 0.03 ± 0.12, suggesting minimal additive genetic effects contribution to the observed phenotypic variation. The additive variance (σ²A) and dominance variance (σ²D) were 8.57 and 229.18, respectively, and the degree of dominance (d²) of 5.15 implies strong overdominance, where homozygotes were outperformed significantly by the heterozygotes. The genetic variance (σ²G) of 237.76 accounted for the majority of the phenotypic variance (σ²P) of 280.20, whereas the environmental variance (σ²E) of 42.45 was moderate (**Table 9**).

Panicle length (PL) exhibited a low narrow-sense heritability of 0.14 ± 0.10, implying that additive genetic effects contribute only modestly to the phenotypic variation of the trait. The additive variance (σ²A) of 2.02 was much smaller than the dominance variance (σ²D) of 11.11, suggesting that non-additive gene action, especially dominance, plays a major role in the inheritance of panicle length. The degree of dominance (d²) of 2.33 indicates overdominance, where heterozygous genotypes may outperform both homozygotes. The genetic variance (σ²G) of 13.15 accounted for the majority of the phenotypic variance (σ²P) of 14.97, whereas the environmental variance (σ²E) of 1.84 was relatively low (**Table 9**).

The number of panicles per plant (NPP) in the F2 rice progenies revealed a very low narrow-sense heritability of 0.07 ± 0.11, suggesting minimal contribution of additive genetic effects to the observed phenotypic variation. The additive variance (σ²A) of 0.614 was much smaller than the dominance variance (σ²D) of 2.075, and the degree of dominance (d²) of 1.82 indicating overdominance, where heterozygous combinations may outperform both homozygotes. The environmental variance (σ²E) of 6.480 was the largest component of the phenotypic variance (σ²P) of 9.175, indicating the strong influence of environmental factors on this trait. The genetic variance (σ²G) of 2.687 was relatively low in comparison, enhancing the conclusion that selection for NPP in early generations may not be reliable (**Table 9**).

Grain length (GL) exhibited moderate narrow-sense heritability of 0.40 ± 0.51, implying that it is influenced by additive genetic effects. The phenotypic variance (σ²P) and genotypic variance of 0.82 and 0.61, respectively, indicate that selection of this trait based on phenotype could be moderately effective. Additive variance (σ²A) of = 0.33 was slightly higher than the dominance variance (σ²D) = 0.28, and the degree of dominance (d²) of 0.94 was close to unity, suggesting partial dominance in the inheritance of grain length. The environmental variance (σ²E) of 0.21 was relatively low, enhancing the idea that genetic factors are the primary contributors to trait variation among the progenies (**Table 9**).

Grain width (GW) in the F2 rice progenies revealed zero (0.00) for narrow-sense heritability (h²) and additive variance (σ²A), suggesting that additive genetic effects were either negligible or absent in the expression of this trait. The dominance variance (σ²D) of 0.08 accounted for the majority of the genetic variance (σ²G) which was also 0.08. The environmental variance (σ²E) of 0.03 was relatively low but still contributed to the total phenotypic variance (σ²P) with a magnitude of 0.12. The degree of dominance (d²) of 0.00 suggests that dominance effects were present but not strong enough to influence the trait in a measurable manner for the present study (**Table 9**).

The grain yield per plant (GYPP) in the studied F2 rice progenies exhibited a moderate narrow-sense heritability of 0.39 ± 0.23, implying that both additive and non-additive genetic effects contribute to the expression of the character. The phenotypic variance (σ²P) and genetic (σ²G) variance were 78.25 and 65.41, respectively, suggesting genetic control of the observed variation. The additive variance (σ²A) of 30.61 and dominance variance (σ²D) of 34.80 were relatively at par, suggesting that both types of gene action are crucial. The degree of dominance (d²) of 1.05 indicates partial to complete dominance in the inheritance of grain yield. The environmental variance (σ²E = 12.84) was lower than the genetic components, reinforcing the potential for effective selection in breeding programmes for this trait (**Table 9**).

### 3.4 Correlation analysis

There were strong positive correlations for such traits as panicle length vs. number of panicles per plant (r = 0.726***), grain length vs. grain yield per plant (r = 0.829***), and grain shape vs. panicle length (0.815***) for the studied F2 progenies. On the contrary, strong negative correlations were obtained in grain width vs panicle length (−0.866***), grain shape vs. grain width (r = −0.966***), and number of days to 50% flowering vs. grain yield per plant (r = −0.493**) as in **Table 10**.

**Table 10:** Phenotypic trait correlation among the F2 progenies.

Trait	DTF 50%	PH	PL	NPP	GL	GW	GS	GYPP
DTF_50%	1							
PH	−0.198	1						
PL	0.461*	0.191	1					
NPP	-0.044	0.087	0.726***	1				
GL	−0.407**	0.845***	0.222	0.118	1			
GW	−0.060	−0.549**	−0.866***	−0.687***	−0.597**	1		
GS	0.094	0.697***	0.815***	0.590**	0.710***	−0.966***	1	
GYPP	−0.493**	0.675**	−0.227	−0.205	0.829***	−0.116	0.274	1

*Significant at p = 0.05, **significant at p = 0.01, ***significant at p = 0.001, DTF 50%, days to 50% flowering; PH, plant height; PL, panicle length (cm); NPP, number of panicles per plant; GL, grain length; GW, grain width; GS, grain shape; GYPP, grain yield per plant.

The traits such as grain length (GL) and plant height) (PH) exhibited strong positive associations with grain yield per plant of (r = 0.829***) and (r = 0.675**), respectively. The number of days to 50% flowering was negatively correlated with grain yield per plant (GYPP) with a magnitude of (r = −0.493**), suggesting early flowering might benefit yield (**Table 10**). Other traits such as grain width (GW), grain shape (GS), and panicle length (PL) exhibited weak or non-significant associations. The grain shape (GS) showed strong positive correlations with panicle length (r = 0.815***), grain length (r = 0.710***) and plant height (r = 0.697***). Negative correlation was also revealed for grain shape with grain width (r = −0.966***) and moderate correlation with number of productive panicles (0.590**). The grain width (GW) exhibited strong negative correlations with panicle length (r = −0.866***), number of productive panicles (r = −0.687***), grain length (r = −0.597**), and plant height (r = −0.549**). These results suggest that wider grains may be associated with shorter plants and panicles, and fewer productive panicles potentially revealing a trade-off in grain morphology and plant architecture. Grain length (GL) further shows a strong positive correlation with plant height (r = 0.845**), moderate negative correlation with days to 50% flowering (r = −0.407**), weak or non-significant correlations with panicle length (PL), and number of productive panicles per plant (NPP). These findings suggest that longer grains tend to be associated with taller plants and earlier flowering, which may have implications for yield optimization and selection in breeding programmes (**Table 10**).

The number of productive panicles (NPP) exhibited a strong positive correlation with panicle length (r = 0.726***) and weak or non-significant correlations with days to flowering and plant height. This suggests that longer panicles are associated with more productive panicles, which may positively influence grain yield. Panicle length shows a moderate positive correlation with days to 50% flowering (r = 0.461*) and weak correlation with plant height (r = 0.191). The implication of these results is that longer panicles may be associated with later flowering, which could influence grain development and yield potential. Plant height (PH) exhibited a weak negative correlation with days to 50% flowering (−0.198) as in **Table 10**.

## 4 Discussion

The number of days to 50% flowering assist in determining the maturity period of the rice crops and early flowering germplasm are usually preferred in areas with an unpredictable weather pattern. The differences in the number of days to 50% flowering among cross combinations in the current study suggest potential genetic influence from parental lines on flowering time, which may impact adaptability and yield performance (**Table 5**). The current results are in agreement with findings reported by earlier workers (Matsubara and Yano, 2018; Pachauri et al., 2020; Mamdouh et al., 2022).

The mean number of panicles per plant for various cross combinations exhibited no significant differences, suggesting that environmental or genetic factors may have influenced the germplasm. Sharma and Jaiswal (2020) conducted combining ability analysis for grain yield and quality traits in Basmati rice, highlighting the role of both additive and non-additive gene action in determining panicle number. These studies have suggested that breeding programmes should focus on optimizing genetic traits that enhance panicle development, corroborating with the current findings.

The significant differences exhibited for plant height among the studied rice germplasm across combinations confirm the presence of diversity (**Table 5**). Majority of the germplasm in the current study were generally described as semi-dwarf, and this is a fundamental trait for lodging resistance which can preserve yield, corroborating with earlier reports by fellow researchers (Pan et al., 2019; Zhang et al., 2019; Shrestha et al., 2020; Wu et al., 2022; Xiong et al., 2022);. Findings of the current study are also in agreement with those reported by fellow workers, where variations in plant height among the rice genotypes were revealed (Pachauri et al., 2020; Owusu et al., 2020; Liang et al., 2021; Liu et al., 2021; Lan et al., 2023). Prabhu and Abhishree (2024) also suggested that plant height is influenced by genetic and environmental factors, which aligns with findings of the present study on variability.

The current study exhibited significant variations among different rice cross combinations for panicle length (**Table 5** and **Figure 2**). Sun et al. (2025) identified elite alleles of enhanced panicle exertion (EPE 1) genes, which significantly increase panicle elongation in rice hybrids. Their findings indicate that genetic modifications targeting gibberellin-related genes can enhance panicle length, supporting the idea that genetic selection plays a fundamental role in optimizing panicle architecture. Other studies by Wang et al. (2024), on mapping of the qPL5 QTL, exhibited that genetic differences in this region significantly influence panicle length. The studies by Sun et al. (2025) and Wang et al. (2024) therefore corroborate with the current findings, emphasizing the genetic basis of panicle length variation. Furthermore, Agata (2025) reviewed the genetic mechanisms influencing diverse panicle architectures in rice germplasm and suggested that QTL pyramiding could be used to enhance panicle length. While Agata (2025) acknowledges the crucial role played by genetic factors, the study suggested that wild Oryza genetic resources could be more effective in improving panicle architecture than traditional hybrid breeding. This perspective is contrary from the current study, which focused on cross combinations involving both landraces and improved cultivars rather than wild genetic resources alone.

Grain size is a critical yield trait influenced by networks of intricate genes, and over 200 genes had been characterized in regulating rice grain size to date. In the current study, moderate variation was exhibited in terms of grain length trait; however, it was statistically insignificant (**Table 5** and **Figure 2**). In their separate evaluations of various hybrid combinations, Kour et al. (2019); Gaballah et al. (2022); Mohanty et al. (2025), and Prapakaran et al. (2025) found moderate to limited variations across hybrids in terms of grain length, corroborating strongly with current findings.

Grain width, length, and thickness (shape) collectively contribute to grain size, which is the main determinant of grain weight and, therefore, overall grain yield. There were statistically significant differences exhibited across cross combinations in the current study, and the mean grain width was 2.5 mm (**Table 5**). These results are in agreement with those reported by previous researchers (Mohanty et al., 2025; Zhang et al., 2025) who found grain width variability across crosses and that genetic backgrounds play a fundamental role in determining this trait.

Grain shape (GS) among the F2 progenies varied across the different cross combinations, with values ranging from 3.2 to 4.3 although not statistically significant at the 5% level (**Table 5**). These results are in agreement with those reported by Anis (2016) and Zhang et al. (2025) who highlighted continuous variation in grain traits across progenies.

Grain yield per plant (GYPP) differed across the studied rice germplasm including the F2 populations; however, analysis of variance exhibited no statistically significant differences (**Table 5** and **Figure 2**). The present findings were corroborating strongly with separate reports by Patel et al. (2025) and Gaballah et al. (2022) who attested to the idea that biological differences in grain yield can be substantial even in the absence of significant variations especially in early segregating generations such as F2s. The biological differences are key for selection in breeding programmes especially in early generation.

General combining ability (GCA) is the average performance of a genotype across multiple hybrid combinations, mainly due to additive genetic effects, whereas specific combining ability is the deviation from expected performance in specific crosses, attributed to non-additive genetic effects such as dominance and epistasis, and is vital for aiding selection of desirable parents and crosses in plant breeding programmes (Veeresha et al., 2015; Abd-El-Aty et al., 2023; Singh et al., 2024). The positive GCA values for the flowering trait suggest that a parent contributes to delayed flowering in progenies, whereas negative values indicate earlier flowering tendencies. The large negative value observed in Kudya may be beneficial for breeding early-maturing varieties, whereas the positive effect of NERICA 4 suggests potential for varieties with extended vegetative growth (**Table 6**). Despite that the Kilombero genotype is well known for late flowering, it might possess recessive (masked) alleles for early flowering that could be inherited by offspring. These results are in agreement with those reported by (Manju et al., 2021; Adjah et al., 2022; Patel et al., 2025) on both positive and negative GCAs.

The findings for plant height GCA reflect how each female parent contributed to this trait in hybrid offspring. A positive GCA value implies a favourable additive genetic effect, whereas a negative value suggests a reduction in plant height. In terms of the female parents used in this study, the MAKAFACI rice variety exhibited a near-neutral effect, suggesting a minimal influence on the inheritance of plant height trait. Apart from Kudya, NERICA 4 also showed a strong negative GCA effect, revealing that this parent significantly leads to a declined plant height in hybrids. This is a fundamental trait in breeding programmes aimed at achieving semi-dwarf rice cultivars, an idle trait for lodging resistance and improved productivity. Findings of the current study are in great agreement with those reported by Devi et al. (2018).

In terms of panicle length, variation in GCA effects was reported among the three male parents suggesting the differential genetic contributions to this trait. The small but positive effect for Kayanjamalo (0.001) implies negligible additive genetic influence on panicle length, meaning it may not significantly enhance this trait in hybrids. Mtupatupa, with a GCA effect of 0.345, presents a relatively stronger additive genetic contribution to panicle length. This means that Mtupatupa could be a favourable male parent in breeding programmes aimed at increasing panicle size, which is often linked to yield potential (**Table 7**).

Conversely, Kilombero exhibited a markedly negative GCA effect indicating that its genetic contribution leads to a declined panicle length. This strong negative magnitude may suggest the presence of alleles that suppress panicle elongation, making Kilombero unsuitable for breeding programmes aimed at increasing panicles length unless combined with a complementary parent possessing high positive GCA effects. Ramakrishna et al. (2024) in their line × tester analysis exhibited that certain parental germplasm had positive GCA effects for panicle length, making them suitable for breeding programmes aimed at enhancing yield traits, corroborating strongly with the present findings. Another study by Zhang et al. (2025) identified alleles such as OsGA2ox9 that negatively regulate panicle elongation, very similar to the suppressive effect seen in Kilombero for the current study.

The present study evaluated grain yield per plant (GYPP) across eight F2 rice cross combinations, revealing moderate variability among progenies. The highest mean yield was observed in the cross Kudya × Kayanjamalo, whereas Uwemi × Kilombero recorded the lowest. Despite the biological variation, the analysis of variance showed no statistically significant differences among the crosses (*F*-prob = 0.19), suggesting that non-additive gene effects had influenced the expression of grain yield (**Table 5**). These findings are in agreement with those of Patel et al. (2025), who reported low heritability for grain yield per plant in an F2 population under drought stress, indicating limited scope for direct selection.

The absence of significant differences for grain yield per plant may also reflect the influence of genotype × environment interactions, as indicated by Singh et al., 2024, who reported variable heterosis and combining ability effects across various rice hybrid combinations. This emphasized the need for multi-environment trials to validate the stability and performance of promising crosses.

The significant variation observed among male parents for grain yield per plant (GYPP) implies the availability of exploitable genetic diversity within this group. This result is in agreement with those reported by Zhang and Bin Rahman (2023), who found that male parental lines in hybrid rice breeding programmes contributed disproportionately to yield gains under both irrigated and rainfed conditions. On the other hand, non-significant differences among female parents and their interactions with male parents suggest a limited combining ability among the female lines used (**Table 6**). Similar patterns were observed by El-Mowafi et al. (2021), who found low general combining ability for yield. However, findings of the present study are in divergence to those of Lipi et al. (2020), who reported significant genetic variability and some promising hybrids. Generally, these findings reinforce the strategic value of selecting genetically diverse and high-performing male lines in early-generation breeding.

Separate studies carried out by Zewdu (2020) and Singh et al. (2019) analysed combining ability in 18 F2 rice populations and heterosis, respectively, and found that parents with high GCA effects significantly influenced grain yield per plant, similar to findings of the current work with Kudya and NERICA 4.

The general combining ability (GCA) effects observed in this study revealed that Kayanjamalo exhibited a negligible but positive contribution to grain yield per plant (0.001 g), whereas Kilombero and Mtupatupa showed negative GCA effects of −7.025 and −6.853, respectively (**Table 7**). These findings suggest that Kayanjamalo may possess favourable additive gene effects for grain yield, making it a promising parent for yield improvement in rice breeding programmes.

Similar trends had been reported in recent studies by Ali et al. (2025), who identified rice lines with positive GCA effects for grain yield per plant, emphasizing the fundamental aspect of selecting parents with strong additive genetic potential for hybrid development. Furthermore, Gitanjali et al. (2025) found that certain testers exhibited significantly negative GCA values, underscoring the variability in parental performance and the need for careful selection in breeding schemes. Other studies by Sameer et al. (2020) also reported contrasting GCA effects among parental lines, strengthening the role of genetic background in determining yield potential. Generally, these findings support the conclusion that identifying and utilizing parents with favourable GCA effects such as Kudya and Kayanjamalo can strengthen the efficiency in early generations advancement for the development of high-yielding rice varieties.

Results of the current study are in agreement with those reported by Zewdu (2020) on combining the ability of selected rice genotypes. In that study, they evaluated 18 F2 populations and found that crosses involving at least one parent with high GCA often exhibited desirable SCA effects, especially for grain yield. This reinforces the current findings where combinations such as Kudya × Kayanjamalo (4.041) can significantly enhance yield (**Table 8**). Similar findings were also reported by fellow researchers (Gupta et al., 2020; Singh et al., 2024; Zhu et al., 2025) who also suggested that selection is more effective in later generations.

Gene action for this study comprised estimation of additive and non-additive genes for selected traits of genotypes. The narrow-sense heritability of 0.24 ± 0.16 obtained in F2 rice progenies of the present study for number of days to 50% flowering indicates the influence of both additive and dominance gene actions (**Table 9**). The equal contribution of additive (σ²A) and dominance variance (σ²D) with the value of 5.58 each, along with a degree of dominance (d²) of 1.00, suggest complete dominance in the inheritance of this trait. The genetic variance (σ²G = 11.20) accounted for nearly half of the phenotypic variance (σ²P = 23.26), whereas the environmental variance (σ²E) of 12.10 was also substantial, indicating moderate environmental influence on heading time. Breeding strategies that combine phenotypic selection with environmental control or marker-assisted selection targeting flowering-related QTLs may enhance genetic gain. The current results are consistent with those by Gupta et al. (2020) who reported high heritability and genetic advance for number of days to flowering in some crosses.

The narrow sense heritability for plant height in the current study was very low (0.03 ± 0.12), suggesting minimal additive genetic effects to the observed phenotypic variation. The additive variance was 8.57, the dominance variance was 229.18, and the degree of dominance was 5.15, and this suggests strong overdominance. The genetic variance (σ²G) of 237.76 accounted for the majority of the phenotypic variance (σ²P) of 280.20, whereas the environmental variance (σ²E) of 42.45 was moderate (**Table 9**). Patel et al. (2025) reported low heritability for plant height in an F2 mapping population attributing the result to dominance gene action and strong environmental influence. Their findings align closely with the current study, reinforcing the limited role of additive effects in early-generation selection. The current findings were also in tandem with those reported by Asati and Yadav (2020) in their earlier study. Breeders should therefore consider exploiting heterosis through hybrid breeding to capitalize on overdominance, or delaying selection to later generations such as F5.

The current study revealed that panicle length (PL) in F2 rice progenies is predominantly governed by non-additive gene action, as evidenced by the low narrow-sense heritability (0.14 ± 0.10) and the high dominance variance (11.11) relative to the additive variance (2.02). The degree of dominance (2.33) shows overdominance, suggesting that heterozygous combinations may outperform both homozygotes for this trait. The genetic variance (13.15) shared a substantial portion of the phenotypic variance (14.97), whereas the environmental variance (1.84) was relatively low (**Table 9**). These results imply that panicle length is best improved through hybrid breeding strategies that exploit heterosis, rather than direct selection in early segregating generations. The present results are in line with those reported by Prapakaran et al. (2025); however, they are contrary to what was revealed by Wang et al. (2024).

The current study revealed that the number of panicles per plant (NPP) in F2 rice progenies is highly influenced by non-additive gene action and environmental factors. The narrow-sense heritability was very low (0.07 ± 0.11), suggesting minimal contribution of additive genetic effects to the phenotypic expression of this trait. This is further supported by the low additive variance (0.614) compared with the dominance variance (2.075), and the degree of dominance (1.82), which shows overdominance. The environmental variance (6.48) was the largest component of the phenotypic variance (9.175), strengthening the idea that NPP is highly sensitive to environmental conditions. The relatively low genetic variance (2.687) further suggests ineffective selection for this trait in early generations and that hybrid breeding strategies for heterosis exploitation may be more appropriate (**Table 9**). The present findings are in agreement with those by Asati and Yadav (2020) and Singh et al. (2020) who separately reported low heritability and genetic advance for number of panicles per plant (NPP) in F2 populations. Bassuony and El-sherbiny (2021) reported that under normal and water-deficit conditions, number of panicles per plant (NPP) showed inconsistent heritability and was influenced by environmental interactions, further supporting the current study.

The present study revealed that grain length (GL) in the F2 rice progenies was governed by both additive and dominance gene actions. The narrow-sense heritability (h²) of 0.40 ± 0.51 exhibits a moderate contribution of additive genetic variance to the total phenotypic variance. The additive variance (σ²A) of 0.33 was slightly higher than the dominance variance (σ²D) of 0.28, and the degree of dominance (d²) of 0.94 was close to unity, showing partial dominance in the inheritance of grain length. The genotypic variance (σ²G) of 0.61 accounted for a substantial portion of the phenotypic variance (σ²P) of 0.82, whereas the environmental variance (σ²E) of 0.21 was relatively minimal (**Table 9**). This suggests that the trait is largely controlled by genetic effects, making it a moderately reliable target for selection. Riyanto et al. (2024) reported that grain length and shape in F2 populations of Basmati × Japonica crosses were controlled by polygenes with high broad-sense heritability and moderate genetic gain, consistent with the current findings’ exhibition of moderate heritability and partial dominance.

Grain width (GW) in the F2 rice progenies of the present study is predominantly influenced by non-additive gene action and environmental effects. The narrow-sense heritability (h²) of 0.00 and additive variance (σ²A) of 0.00 reveal a complete absence of additive genetic effects, suggesting that selection based on phenotypic expression in early generation would be ineffective. The dominance variance (σ²D = 0.08) accounted for the whole of the genetic variance (σ²G = 0.08), whereas the environmental variance (σ²E = 0.03) had a small contribution to the phenotypic variance (σ²P = 0.12) (**Table 9**). The degree of dominance (d²) of 0.00, despite the presence of dominance variance, suggests that dominance effects were not strong enough to influence the trait in a predictable manner. These findings indicate that grain width is under complex genetic control, such as epistatic interactions or polygenic dominance, and is highly sensitive to environmental conditions. Selection for this trait in early generations may therefore not be effective, and the recent results strongly agree with those reported by Asati and Yadav (2020) and Ponce et al. (2020).

The present study revealed that grain yield per plant (GYPP) in the F2 rice progenies is governed by both additive and non-additive gene actions. The narrow-sense heritability (h²) of 0.39 ± 0.23 indicates a moderate contribution of additive genetic variance to the total phenotypic variance, implying that selection should be complemented by strategies that also consider dominance effects. The additive variance (σ²A) of 30.61 and dominance variance (σ²D) of 34.80 were closely at par, and the degree of dominance (d²) of 1.05 suggests partial to complete dominance (**Table 9**). This suggests that heterozygous combinations may have yield advantage, reinforcing the potential for exploiting heterosis in breeding programmes. The genotypic variance (σ²G) of 65.41 was substantial relative to the environmental variance (σ²E) of 12.84, suggesting that there is a genetic control of the observed phenotypic variation. The current findings are in tandem with those reported by Asati and Yadav (2020). They observed high heritability with low genetic gain for grain yield per plant in F2 progenies, showing the presence of non-additive gene action and dominance effects.

Correlation analysis studies the joint variation of two or more variables for determining the amount of association between those variables, Kothari and Garg (2020). There were positive significant correlations exhibited between plant height (PH) and grain length (GL) (r = 0.845***) suggesting that taller plants may produce longer grains, which could be ideal for certain consumer preferences. Negative correlations such as number of days to flowering (DTF_50%) and grain yield per plant (GYPP) (r = −0.493**) also suggest that early flowering may enhance yield, a valuable trait for terminal drought escape where rainfall is erratic (**Table 10**).

Furthermore, grain yield per plant (GYPP) showed strong positive significant correlation with grain length (GL) (r = 0.829***), revealing that grain length is a major contributor to yield. On the other hand, panicle length (PL) and number of panicles per plant (NPP) had negative or weak correlations with grain yield per plant (GYPP), indicating that indirect selection may be more effective. The significant negative correlation between grain width (GW) and grain size (GS) (r = −0.966***) suggests a trade-off that breeders must navigate when advancing germplasm for grain shape. Traits like GS and GL are positively correlated with GYPP, making them suitable targets for selection (**Table 10**). The implication of these relationships means that grain shape is tightly linked to both panicle and grain morphology, which may influence overall yield potential. The findings of the current study are in line with those by Pance et al., (2020), Swapnil and Manigopa (2020), who reported a strong positive correlation between grain length and grain yield. Manoj et al. (2023) in their study on phenotypic correlation in F2 rice populations reported a positive association in plant height, panicle length, and grain yield, supporting the recent findings on plant height, grain length, and grain yield per plant relationships. On the contrary, Carsonol et al. (2023) worked on drought-related traits such as heading date and spikelet fertility, some traits exhibited epistatic inheritance, which explains weak association with the recent findings on such traits.

## 5 Conclusion

Findings of the present study identified the female and male parents such as Kudya and Kayanjamalo, respectively, with the potential to produce superior hybrids owing to their high and moderate GCA effects on grain yield. The superior hybrid combinations that recorded the highest grain yield per plant was exhibited in Kudya and Kayanjamalo genotypes. Traits such as number of days to flowering, grain yield per plant, and grain length, although not very high magnitude, displayed moderate heritability of 0.24, 0.39, and 0.40, respectively, suggesting possible inheritance of these traits. Genetic analysis revealed that plant height for NERICA 4 and Kudya had a strong negative GCA suggesting their potential for producing semi-dwarf cultivars which can resist lodging. There were positive correlations exhibited between plant height (PH) and grain length (GL), and grain yield per plant (GYPP) with grain length suggesting that taller plants may produce longer grains. Breeding programmes aimed at increasing grain length and shape can use MAKAFACI and Kilombero genotypes, respectively, as suitable parents owing to the positive GCA effects for these traits. Future studies for the advancement of filial generation should incorporate the use of molecular markers to complement phenotypic evaluation involving combining abilities, gene action, and heritability for the target traits. Findings of the current study are useful to rice breeders who want to develop cultivars to meet different breeding objectives, such as grain yield and good quality.

## Data Availability

The datasets presented in this study can be found in online repositories. The names of the repository/repositories and accession number(s) can be found in the article/**Supplementary Material**.
